# Knowledge and attitudes of non-occupational HIV post-exposure prophylaxis amongst first- and second-year medical students at Stellenbosch University in South Africa

**DOI:** 10.4102/phcfm.v6i1.665

**Published:** 2014-11-24

**Authors:** Nondumiso B.Q. Ncube, Willem A.J. Meintjes, Lumbwe Chola

**Affiliations:** 1 Division of Community Health, Stellenbosch University, South Africa; 2 Unit for Infection Prevention and Control, Stellenbosch University and Tygerberg Hospital, and Division of Community Health, Stellenbosch University, South Africa; 3 Health Systems and Services Research, Division of Community Health, Stellenbosch University, South Africa

## Abstract

**Background:**

Human immunodeficiency virus (HIV) infection is a worldwide problem, with 68% of infected people residing in sub-Saharan Africa. Antiretroviral therapy is used as post-exposure prophylaxis (PEP) to prevent infection in cases of occupational exposure, and use has recently been expanded to non-occupational exposure. Studies have demonstrated a lack of awareness of non-occupational PEP (NO-PEP) in the general population.

**Aim:**

The aim of this study was to evaluate knowledge and attitudes towards availability of, access to and use of NO-PEP amongst first- and second-year medical students.

**Setting:**

Participants were medical undergraduates of Stellenbosch University in the Western Cape of South Africa who were registered in 2013.

**Methods:**

A descriptive cross-sectional study of 169 students was performed. Data were collected using self-administered questionnaires handed out in a classroom in August 2013. Self-reported knowledge and attitudes towards NO-PEP and barriers to access to and use of NO-PEP were analysed using frequency tables. Associations between self-reported and objective knowledge of NO-PEP were analysed by odds ratios.

**Results:**

Over 90% of students had good knowledge on HIV transmission, and about 75% knew how it can be prevented. Twenty eight per cent (*n* = 47) of students reported knowledge of NO-PEP; 67% reported hearing about it from lecturers, whilst 1% reported hearing about it from their partner. Students who knew the correct procedure to take when a dose is forgotten were 2.4 times more likely to report knowledge of NO-PEP than those who did not know what to do when a dose is forgotten (*p* = 0.029). No other associations were statistically significant.

**Conclusion:**

Students had positive attitudes towards the use of NO-PEP and also identified barriers to its use. Despite good knowledge of HIV prevention and transmission, knowledge on NO-PEP was poor.

## Abstrait

**Contexte:**

**Connaissances et Attitudes de la Prophylaxie non-professionnelle post-exposition au VIH chez les étudiants en médecine de première et seconde année à l'université de Stellenbosch en Afrique du Sud**.

**Objectif:**

Le virus de l'immunodéficience humaine (VIH) est un problème mondial avec 68% des personnes infectées résidant en Afrique sub-saharienne. La thérapie antirétrovirale est utilisée comme prophylaxie post-exposition (PEP) pour prévenir l'infection due à l'exposition professionnelle, et son utilisation a récemment été étendue aux expositions non professionnelles. Des études ont révélé un manque de sensibilisation de la PEP (NO-PEP) non professionnelle parmi la population en général. Le but de cette étude était d’évaluer les connaissances et attitudes envers la disponibilité, l'accès et l'utilisation des NO-PEP chez les étudiants en médecine de première et seconde année.

**Méthodes:**

Une étude transversale et descriptive de 169 étudiants a été effectuée. On a collecté des données à l'aide de questionnaires auto-administrés distribués dans une salle classe en août 2013. Les connaissances déclarées et les attitudes envers le NO-PEP, ainsi que les barrières à l'accès et à l'utilisation du NO-PEP ont été analysées au moyen de tableaux de fréquence. Les associations entre les connaissances déclarées et les connaissances objectives du NO-PEP ont été analysées par rapports de risques.

**Résultats:**

Plus de 90% des étudiants avaient de bonnes connaissances sur la transmission du VIH, et environ 75% savaient comment la prévenir. Vingt-huit pour cent (*n* = 47) des étudiants ont déclaré connaitre le NO-PEP. Soixante-sept pour cent des étudiants ont dit avoir eu connaissance du NO-PEP par leurs professeurs alors que 1% ont dit que leur partenaire leur en avait parlé. Les étudiants qui connaissaient la procédure correcte à suivre quand on oublie de prendre une dose, avaient 2.4 plus de chance de signaler leur connaissance du NO-PEP que ceux qui ne savaient pas quoi faire quand on oublie une dose (*p* = 0.029). Aucune autre association n'avait d'importance du point de vue statistique. Les étudiants avaient des attitudes positives envers l'utilisation du NO-PEP et pouvaient aussi identifier les barrières à son utilisation.

**Conclusion:**

Malgré une bonne connaissance de la prévention et de la transmission du VIH, la connaissance du NO-PEP était mauvaise.

## Introduction

### HIV infection in sub-Saharan Africa and South Africa

The human immunodeficiency virus and/or acquired immunodeficiency syndrome (HIV/AIDS) epidemic continues to be a problem several decades after it was first discovered. According to the World Health Organisation (WHO) update on the global AIDS epidemic, ‘34 million people were living with HIV at the end of 2010’.^1^ HIV infection is spreading at a fast pace, with over 2.7 million infections each year, and sub-Saharan Africa bearing most of these: ‘In 2010, about 68% of people living with HIV in the world were residing in sub-Saharan Africa’.^1^ In July 2008 the Joint United Nations Program on AIDS-WHO estimated that the number of people living with HIV infection in South Africa aged between 15 and 49 years was 5.3 million,^2^ with the Western Cape province prevalence ranging from 1% to 4.9%.^9^

### Antiretroviral therapy

There are several ways in which HIV infection can be prevented and treated. For treatment, the WHO recommends antiretroviral therapy (ART). ART is the use of a combination of antiretroviral (ARV) medicines taken orally to suppress HIV infection by controlling replication of the virus within the infected individual's body.^1^ HIV makes the host's immune system weak, and hence the person is unable to fight infections. The use of ARV medicines therefore strengthens the immune system and helps it to regain the power to fight off infections. In South Africa the use of ARVs began in 2003,^3^ and the WHO ‘recommends that adults infected with HIV initiate ART at CD4^**+**^ cell counts of **≤** 350 cells/µL’.^3^

First-line ART comprises a backbone of two nucleoside and/or nucleotide reverse transcriptase inhibitors (NRTIs, such as zidovudine, abacavir or tenofovir; plus lamivudine or emtricitabine); and a non-nucleoside reverse transcriptase inhibitor (NNRTIs, either nevirapine or efavirenz).^4^ For second-line treatment the WHO Rapid ADVICE Guidelines recommend the use of two NRTIs (tenofovir plus lamivudine/emtricitabine, or zidovudine plus lamivudine) as the backbone together with a ritonavir-boosted protease inhibitor such as lopinavir or atazanavir.^5^

Amongst the various prevention measures for HIV, ART is recommended particularly in emergency situations. ART is mainly used by medical personnel after exposure to HIV-infected tissues and fluids. Recently the use of ART to prevent infection post non-occupational exposure to HIV has increased, with most countries developing guidelines for this. Clinical trials that prove the effectiveness of using ART to prevent HIV infection have not been carried out due to ethical reasons.

### Post-exposure prophylaxis

First-aid is given post-occupational exposure to HIV-infected tissues or fluids, followed by emergency ART. The reason for giving first-aid before putting the individual on emergency ART is to lessen the time of contact with the infected bodily fluids and tissues, hence reducing the risk of infection. In situations where the skin is cut, the site is washed with soap and water and the wound is encouraged to bleed freely under running water for several minutes or until bleeding ceases, with no use of strong agents like alcohol. If there is no water, the area is cleaned with available gel or solution for cleaning hands. If, however, there is a splash with body fluids or blood and the skin is intact, the same procedure mentioned above is followed and use of a mild disinfectant like 2% – 4% chlorhexidine gluconate on the site is encouraged.^6^

The use of ART for occupational post-exposure prophylaxis (PEP) and its expansion for use in non-occupational exposures has caused a lot of uncertainty for providers of health care and policy makers.^5^ Non-occupational exposure to HIV happens where an individual is exposed to the virus in ways other than whilst executing their professional duties. This could be the case where there is substantial risk of acquiring HIV infection when body openings such as the eye, vagina, mouth, rectum and/or other mucous membranes or non-intact skin are contaminated with any infected body fluids when the contaminating source is HIV positive.

Key issues amongst provision of non-occupational PEP are ‘the appropriate indications for HIV-PEP, ART choices, and management strategies to accompany the use of PEP for HIV’.^5^ As there is expansion in the use of ART, policy decisions and plans for delivering services even to settings that have poor resources are required in implementing ART programmes.

Depending on the severity of exposure, the exposed individual may take a combination of two drugs (zidovudine or tenofovir disoproxyl fumarate, and lamivudine) if the risk of exposure is low; or three drugs (zidovudine or tenofovir disoproxylfumarate, lamivudine and lopinavir/ritonavir) for high- risk exposure.^7^ High-risk exposure to HIV is when a HIV-negative person comes into contact with the blood of somebody who is infected, tissues and body fluids where there is intramuscular injury, injury is caused by a device entering a blood vessel, injury with a hollow-bore needle, as well as when the mucous membranes of an uninfected person come into contact with the above-mentioned products from an infected person. The risk is also considered high if the infected person's viral load is high.^7^

Low-risk exposure to HIV, on the other hand, is when the skin of the HIV-negative person is not broken when it comes into contact with the infected person's tissues, blood and body fluids.

## Literature review

This section discusses the literature on non-occupational PEP, including guidelines, knowledge, and its use. Non-occupational PEP for HIV infection is defined as ‘a course of antiretroviral drug treatment taken for the prevention of HIV infection after a potential non-occupational exposure to the virus for example after unprotected sexual contact or sharing injecting drug equipment with an HIV positive person.’^8^

An extensive search was performed on Medline, Scopus, the WHO website and Google Scholar using the search string (awareness or knowledge or attitudes or beliefs) and (non-occupational HIV) and (post-exposure prophylaxis). The initial search provided about 950 articles, and 22 were used for the study. Each article was examined by reading its abstract, opening paragraph and conclusion and a general browse of the whole article to see the relevance of the material to this study. A working bibliography was hence generated. Then each of the articles was thoroughly investigated in terms of the depth and breadth of coverage of the topic to ascertain whether they were pertinent to this research. After deciding on the sources that were relevant, the quality of each article was assessed to see if the arguments presented were clearly stated, well researched and reasonable. Sources were also chosen on the basis of how old the information was and whether up to date and accurate in the field on non-occupational PEP. The search was not restricted by date, study design or language. However, only articles published in English were used. The following key words were used: human immunodeficiency virus, non-occupational PEP, knowledge, awareness, attitudes, beliefs, practices.

### Guidelines on use of non-occupational post-exposure prophylaxis

Guidelines on provision of occupational and non-occupational PEP were developed by the International Labour Organisation and WHO in 2005 and became available in 2006.^5^ These guidelines address healthcare workers’ needs and non-healthcare workers who are exposed to blood and/or body fluids infected with HIV.

The guidelines on use of non-occupational PEP differ from one country to another. The National Australian PEP guidelines recommend that the exposed person be initiated on a four-week course of treatment,^8^ and an HIV test must be done before initiation (within 72 hours of exposure). The HIV test must be repeated at ‘4 weeks, 3 months and 6 months after commencement of non-occupational PEP.’^8^ This is in line with the WHO recommendations, which state that the exposed individual must be initiated on a 28-day course of ART within 72 hours of exposure, with follow-up care.^1^

South Africa also has guidelines recommending the use of PEP after sexual exposure to HIV which are similar to the WHO and Australian PEP guidelines.^9^ However, there are few published studies that monitor follow-up of patients to completion of PEP. The first pioneering PEP provision initiatives in South Africa began around 1998, before the national PEP policy or guidelines were adopted.^10^ However, without a transparent national directive with regard to PEP, these initiatives were not implemented successfully.

In 1999 the South African Medical Association released a statement which ‘supported PEP provision for rape survivors, and subsequently publicised guidelines on support to rape survivors in liaison with the Minister of Health, local and international non-governmental organisations as well as the pharmaceutical industry.’^10^ The South African Cabinet finally announced that it supported the provision of PEP for people that have been raped who attend South African healthcare facilities in 2002.^10^ The PEP followed protocols established by other developing countries, which comprised a two-drug regimen of zidovudine and lamivudine.^10^

In countries where there has been implementation and use of official recommendations for non-occupational PEP, there has been a change in practitioners’ attitudes towards non-occupational PEP as well as an increase in the number of prescriptions.^11^

### Knowledge of post-exposure prophylaxis

Knowledge of PEP in the general population is quite scant. The topic has not been studied to a great extent, and the few studies that have been done highlight the magnitude of this lack of awareness. In a study of 2932 French people living with HIV infection with a median age of 40 years, a number of factors were found to be associated with no knowledge of PEP.^12^ These were a CD4 cell count of below 200, low educational level, older age, and unemployment.^12^ Of the 2932 participants recruited, 2280 were sexually active. Amongst sexually active participants, 16% were immigrants, 26% were females, and 41% were homosexual men. Homosexual men were more aware of PEP compared to the rest of the participants: ‘Awareness was significantly higher in sexually active people (69.7% versus 52.6% in non-sexually active respondents; *p*= 0.001).’^12^ Individuals who had used injectable drugs in the previous 12 months accounted for 2.1% of the whole sample; in this group PEP awareness was not significantly different in both sexually active and inactive intravenous drug users (66.7% versus 60.9%; *p*= 0.645).^12^

A study that sought to understand factors associated with knowledge of non-occupational PEP and pre-exposure prophylaxis (PrEP) in high-risk men who have sex with other men in New York City, found that these men had a low level of knowledge:

Ethnicity, previous HIV testing, gay self-identification, higher educational level, having a primary provider aware of sexual orientation, reported interaction with healthcare system, and use of internet to meet sex partners were each significantly associated with awareness of PEP or PrEP.^13^

In the United Kingdom there has been a demonstrated increase in knowledge and awareness of non-occupational PEP. A study on nurses working in sexual health clinics showed that 72% (from a sample of 402) had experience of its use in their clinic, and 21% indicated existence of a specific HIV non-occupational PEP policy in their clinic.^14^ Another study in the United Kingdom that assessed clinical practice and opinions on non-occupational PEP found that the number of prescriptions increased between 1997 and 1999. The study attributed this increase to a mix of factors, such as increasing public and physician awareness, increased risky sexual behaviour, as well as improved access to PEP.^15^

The findings of a study done in Western Australia showed that level of awareness increased after a multimodal communication strategy on PEP, and the need for ongoing activities to raise awareness on non-occupational PEP was identified.^8^

### Use of non-occupational post-exposure prophylaxis

A few studies have been done on non-occupational PEP, and the use of medication to prevent HIV transmission in non-occupational exposure is still relatively new in South Africa. A study based on a review of literature on sexual violence and the use of PEP following occupational and non-occupational exposures was conducted in South Africa.^10^ The study incorporated perspectives from in-depth interviews of people who had been raped, activists in HIV and gender, non-governmental domestic violence organisations, rape crisis centres, physicians, lawyers, researchers, and HIV and/or AIDS advisors in the Department of Health.^10^ The study found that:

… the public health and social justice rationale for implementing PEP was equal and actually exceeded industrialised countries. However, issues of delays in accessing PEP caused by the public justice system and lack of training for service providers were found to be significant obstacles to effective implementation.^10^

The increase in uptake of non-occupational PEP in many countries can be attributed to survivors of sexual assault being referred to specialised local clinics until the PEP treatment course is complete, and these people being followed up after initiation.^16^ However, there still are challenges in ensuring effective uptake, particularly in low- and middle-income countries, where there usually is no formal way to track patients within and between such clinics, along with lack of an approach to ensure that patients complete their treatment.^16^ A study in a rural hospital in South Africa showed that ‘sexual assault survivors were three times more likely to complete the entire PEP course when they received care from specially trained nurses.’^17^

Although some countries have guidelines and recommendations in place, there still are conflicting attitudes and practices amongst physicians.^18^A factor that could be affecting the prescribing and uptake of non-occupational PEP is that some medical practitioners ‘do not view rape as a serious medical condition’^16^ A cross-sectional study on nurses and doctors in South Africa showed that ‘a third of these practitioners did not view rape as a serious medical condition, and less than a third of them had been trained on caring for rape victims’.^16^ Furthermore, around 60% of the practitioners reported that the facilities in which they worked had no protocol for rape care, and about half of the practitioners had referred rape survivors for counselling.^16^

### Justification of the study

Studies on awareness of PEP are scant, but have been done in the United Kingdom, Mumbai in India and Nigeria, mainly focusing on assessing occupational exposure. These studies found that awareness was low amongst surgical residents and doctors (around 40%),^19,20^ and higher in nurses (around 72%).^14^ Studies on social exposure have been done in New York City on high-risk men who have sex with men,^13^ and in California on homosexual and bisexual men.^21^ Generally, there is a lack of literature on PEP and its use, particularly in low- and middle-income countries.

In South Africa non-occupational PEP has mainly been used in cases of sexual violence. There is little published literature documenting its use and the knowledge that both patients and health practitioners have with regard to non-occupational PEP. This is particularly worrying, since the studies have shown that rape is not viewed as a medical condition requiring critical care by most medical professionals, and that many of them lack training on how to give proper medical and supportive treatment to rape survivors.^16^ Also, information gathered from Tygerberg Hospital casualty department in the Western Cape showed that few people are initiated on non-occupational PEP, and a larger number is initiated on occupational PEP. Apparently there is no functional system that ensures that the people initiated on PEP are referred to specialised clinics for supportive care that will ensure that they complete their course of treatment. Hence little is known about treatment completion and follow-up. As the country continues to contain the HIV and/or AIDS epidemic, this issue is of concern because it could help to reduce new infections.

This exploratory study was performed to provide baseline information to fill the gap on knowledge and attitudes towards NO-PEP. This study aimed to evaluate knowledge and attitudes towards the availability of, access to and use of NO-PEP at Tygerberg medical campus in South Africa amongst aspiring medical professionals. The secondary objectives were to describe barriers to the access and use of NO-PEP, and to evaluate the association between specific knowledge items and self-reported knowledge of NO-PEP.

Medical students were chosen as the target population for this study because they are future health workers and are at risk of both occupational and non-occupational HIV infection. Ensuring that they are equipped with the necessary information on what NO-PEP is and how to access it in cases of exposure is critical in sustaining human resources in the health sector. The university is a relatively closed community. However, information gathered from the students could provide insight into the knowledge and attitudes on NO-PEP from a wide range of individuals with varying backgrounds, cultures and ethnicities, although participants were limited to only first- and second-year medical students.

## Research methods and design

An exploratory cross-sectional descriptive study was performed in August 2013 at the medical campus of Stellenbosch University in Cape Town, South Africa, after obtaining ethics approval from the Stellenbosch University Health Research Ethics Committee (reference S13/06/119). The medical campus is situated at Tygerberg Hospital, a teaching hospital for Stellenbosch University Faculty of Medicine and Health Sciences.^22^ Tygerberg Hospital is a tertiary Government-owned facility located in Parow, about 20 km from Cape Town city centre; ‘It is the largest hospital in the Western Cape and the second largest hospital in South Africa.’^22^According to Stellenbosch University Faculty of Medicine and Health Sciences: ‘The Faculty of Medicine has approximately 3000 full-time undergraduate and postgraduate students, and each year produces an average of 160 doctors, 60 specialists and 180 graduates and diplomates in supplementary health sciences.’^23^ Tygerberg campus provides accommodation for about 687 undergraduate students. In 2013 the Faculty had 1522 medical undergraduates (254 in year 1; 298 in year 2; 257 in year 3; 235 in year 4; 174 in year 5; 191 in year 6; 56 in the first-year extended degree programme; and 57 in the second-year extended degree programme).

Participants were included if they were medical undergraduates of Stellenbosch University registered in 2013, attended the lecture in which the questionnaire was administered, and if they agreed to participate in the study. The questionnaire used for this study was adapted from a questionnaire used in a previous study.^2^ The researchers modified some of the questions to meet the objectives of the present study. The questionnaire was piloted in a group of Master's in Clinical Epidemiology students before finally being approved by Stellenbosch University Health Research Ethics Committee.

The researchers consulted the timetables for the medical students (year 1 to year 6). Conveners for modules were then requested to allow researchers to administer the questionnaires prior to the start of any lecture on the timetable. Questionnaires were administered on the basis of a convenor agreeing to give some time of their lecture for the researchers to administer the questionnaire. A sample of 197 students was obtained, with 47% in year 1, 40% in year 2, 2.1% in year 3, no fourth year students, 1% in year 5, and 6.7% in year 6.

Students were given information on the study and asked to fill in self-administered questionnaires prior to the start of a class lecture scheduled on their timetables. The questionnaires were handed back to the investigator immediately after completion. Only persons who did not attend the class, those refusing participation, and those with mostly incomplete responses to questions were excluded. Data were carefully captured into an Excel spreadsheet by the primary investigator, and analysed using Microsoft Excel and Stata (version 12.1). Range and consistency checks were used to ensure data quality and minimisation of errors.

Knowledge and attitudes of students towards NO-PEP were assessed by means of frequency tables. Barriers to the use of NO-PEP were also assessed using frequency tables. Associations between self-reported knowledge and selected knowledge questions (objective knowledge) on PEP and NO-PEP were explored using Pearson's chi-square tests for normally distributed data and Fisher's exact tests for non-normally distributed data. Crude odds ratios with 95% confidence intervals were also calculated for the associations between objective and subjective knowledge of PEP and NO-PEP. Persons were excluded from the analysis if either of the questions being evaluated (i.e. subjective or objective knowledge questions) was left blank.

## Results

One hundred and ninety seven students participated in the study. Data from the first- and second- year groups (*n*= 169) were considered representative samples due to low response rates in the other study years (despite efforts to increase participant numbers). Students from third year upwards were busy with clinic rotations and not attending formal lectures at Tygerberg campus. Efforts were made to administer questionnaires to those that had lectures on campus, but these numbers were too low. The researchers tried to administer questionnaires during the students’ clinic visits, but it was not possible for students to leave patients unattended to answer the questionnaires. Therefore respondents from third year and above as well as those who submitted questionnaires with the majority of questions incomplete were excluded from the analysis (*n* = 28).

Just over half of the analysed sample was first-year students (53.8%), whilst 46.2% were in their second-year of study. The mean age of the 169 respondents was 19.7 years (standard deviation 1.9 years) and the range was 17–36 years. There were 40 males and 128 females, with one person not indicating their gender. Students living on campus comprised 53.8% (*n* = 91) of the sample, and those living off campus 45.6% (*n* = 77). One person did not indicate where they lived. Forty one per cent of the students were white, 30% Mixed race, 15% African, 11% Indian and 3% did not indicate their race.

Table 1 summarises the responses that were given on HIV transmission and prevention knowledge items. Only 27.8% of the students reported that NO-PEP can be used to prevent HIV infection.

**TABLE 1:** Participants’ HIV transmission and prevention knowledge.

Participants knowledge	Sources	Yes		No		Unsure		Excluded	
			
		*n*	%	*n*	%	*n*	%	*n*	%
Knowledge on how HIV can be transmitted	Sexual contact	162	95.9	7	4.1	0	0.0	0	0.0
	Mother to child	160	94.1	8	4.7	1	0.6	0	0.0
	Blood transfusion	161	95.3	5	3.0	3	1.8	0	0.0
	Skin cuts	155	91.7	11	6.5	3	1.8	0	0.0
	Needle sharing	163	96.4	6	3.6	0	0.0	0	0.0
	Kissing	9	5.3	148	87.6	10	5.9	2	1.2
Knowledge on how HIV can be prevented	Abstinence	166	98.2	1	0.6	1	0.6	1	0.6
	Being faithful to one partner	151	89.3	11	6.5	4	2.4	3	1.8
	Condom use	151	89.3	10	5.9	7	4.1	1	0.6
	Showering after sex	5	3.0	161	95.3	1	0.6	2	1.2
	ART prophylaxis	125	74.0	12	7.1	26	15.4	6	3.6
Specific knowledge items	Know occupational PEP is used to prevent HIV infection	129	76.3	30	17.8	4	2.4	6	3.6
	Know NO-PEP is used to prevent infection	47	27.8	91	53.8	22	13.0	9	5.3

### Non-occupational post-exposure prophylaxis

**FIGURE 1:**
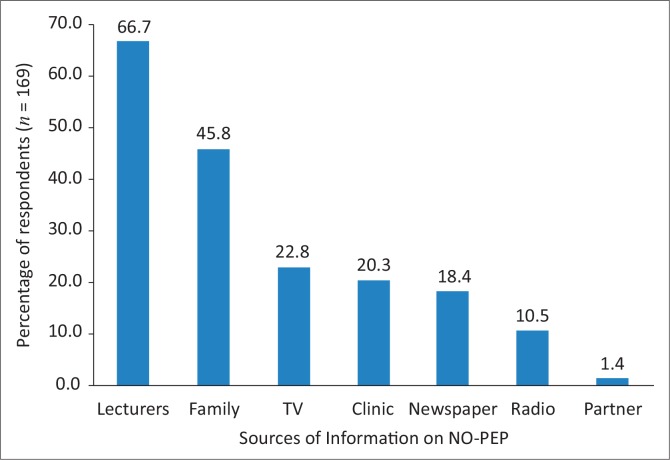
Sources of information on NO-PEP. NO-PEP, non-occupational post-exposure prophylaxis.

Sixty seven per cent of participants reported that they had heard of NO-PEP from lecturers, whilst 1.4% had heard about it from a partner (Figure 1).

In determining students’ attitudes towards NO-PEP, it was found that 66% felt that it is important for people to be given NO-PEP, yet a small proportion (27.8%) reported knowledge thereof. Eighty nine per cent of students felt there were benefits in learning about NO-PEP. Table 2 highlights circumstances under which students thought NO-PEP should be given and those under which NO-PEP is beneficial.

**TABLE 2:** Attitudes towards non-occupational post-exposure prophylaxis.

Attitudes	Item	Yes		No		Unsure		Excluded	
			
		*n*	%	*n*	%	*n*	%	*n*	%
Would take ART if they thought they were exposed to HIV	-	144	85.2	6	3.6	7	4.1	12	7.1
Students’ reported attitudes regarding making NO-PEP available under the following circumstances	Rape	156	92.3	2	1.2	2	1.2	9	5.3
	Sharing razors	74	43.8	58	34.3	28	16.6	9	5.3
	Sharing piercing objects	110	65.1	36	21.3	14	8.3	9	5.3
	Sharing needles	125	74.0	28	16.6	7	4.1	9	5.3
	Having unprotected sex	111	65.7	38	22.5	11	6.5	9	5.3
Students’ reported attitudes regarding benefits of learning about NO-PEP	Learning about NO-PEP is beneficial	151	89.3	10	5.9	2	1.2	6	3.6
Reported benefits of learning about NO-PEP	To prevent HIV infection	120	71.0	15	8.9	18	10.7	16	9.5
	To seek early medical help	131	77.5	4	2.4	15	8.9	19	11.2
	To protect partner	133	78.7	6	3.6	13	7.7	17	10.1
	To protect unborn baby	133	78.7	7	4.1	12	7.1	17	10.1
	Proxy diagnosis of partner	76	45.0	16	9.5	57	33.7	20	11.9

NO-PEP, non-occupational post-exposure prophylaxis; ART, antiretroviral therapy.

**FIGURE 2:**
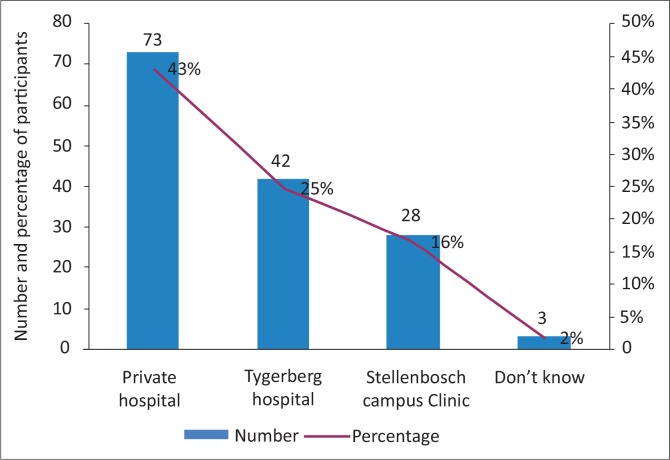
Where students would go to access PEP services. PEP, post-exposure prophylaxis.

In assessing barriers to accessing NO-PEP, although students chose more than one option a large proportion reported that they would access NO-PEP services from a private hospital or doctor, whilst a few did not know where such services could be accessed (Figure 2).

Students also identified barriers to the use of and access to PEP (Table 3).

**TABLE 3:** Barriers to use of and access to non-occupational post-exposure prophylaxis.

Item	Yes		No		Unsure		Excluded	
			
	*n*	%	*n*	%	*n*	%	*n*	%
Rejection by family.	80	47.3	52	30.8	30	17.8	7	4.1
Being neglected by partner.	108	63.9	32	18.9	22	13.0	7	4.1
Being neglected by friends.	102	60.4	36	21.3	24	14.2	7	4.1
Discrimination by healthcare workers.	91	53.8	50	29.6	21	12.4	7	4.1
Discrimination by friends.	121	71.6	23	13.6	18	10.7	7	4.1
Discrimination by colleagues.	115	68.0	29	17.2	18	10.7	7	4.1
Discrimination by employers.	115	68.0	26	15.4	21	12.4	7	4.1
Discrimination by family.	112	66.3	29	17.2	20	11.8	8	4.7
Accessibility of PEP services.	84	49.7	28	16.5	47	27.8	10	5.9
Ashamed to discuss condom use.	10	5.9	148	87.6	5	3.0	6	3.6
Ashamed to discuss not sharing needles.	4	2.4	157	92.9	2	1.2	6	3.6
Ashamed to discuss not doing drugs.	3	1.8	158	93.5	2	1.2	6	3.6
Ashamed to discuss going for HIV test.	11	6.5	147	87.0	5	3.0	6	3.6

PEP, post-exposure prophylaxis.

There was a high non-response rate for questions relating to knowledge on medication to be taken for NO-PEP and precautions to be taken whilst on the medicines. For those who answered these questions, when comparing objective (assessed via specific knowledge questions) and subjective (self-reported) knowledge about NO-PEP, the associations shown in Table 4 were observed. Respondents who knew the correct procedure to take when a dose is forgotten were 2.4 times more likely to report knowledge of NO-PEP than those who did not know what to do when a dose is forgotten (*p* = 0.029). No other associations were statistically significant.

**TABLE 4:** Associations between subjective and objective knowledge of non-occupational post-exposure prophylaxis.

Item	*n*	%	OR 95%	CI	*p*-value	Excluded†	

						*n*	%
Know HIV can be prevented with medication.	55	35.3	1.9	0.9–3.8	0.079	60	35.3
Know how PEP should be taken.	91	71.7	2.1	0.8–5.4	0.105	81	47.6
Know how long PEP should be taken.	46	37.1	1.7	0.8–3.7	0.183	83	48.8
Know what to do in case of a forgotten dose.	38	31.1	2.4	1.1–5.4	0.029	86	50.6
Know to practice safe sex.	116	93.5	3.3	0.4–27.6	0.433	84	49.4
Know to refrain from blood donations.	102	82.3	1.2	0.4–3.4	0.705	84	
Know not to share razors, toothbrushes, needles.	115	92.7	3.8	0.5–31.5	0.273	84	49.4
Know to avoid pregnancy.	80	64.5	2.2	0.9–5.2	0.068	84	49.4
Know to stop breastfeeding.	85	68.5	1	0.4–2.3	0.984	84	49.4

† Participants who did not answer both of the questions.

PEP, post-exposure prophylaxis; NO-PEP, non-occupational post-exposure prophylaxis.

## Discussion

This exploratory study was conducted amongst first- and second-year students at Stellenbosch University to determine knowledge and attitudes towards NO-PEP. The results indicate low reported levels of knowledge of NO-PEP in this population. This is possibly because the topic is not given enough attention in schools and communities, but this assumption needs to be explored further. Although NO-PEP knowledge was low, students reported good knowledge on HIV transmission and the modes of preventing transmission. This shows the need to increase awareness through the clinic (via increased educational campaigns) and mass media (especially radio, TV, and newspapers, as people listen to, watch and read these). This lack of knowledge on NO-PEP is in line with the findings of other studies.^12,13,18,19,20,21^

Most students reported that they would take ART if they thought they had been exposed to HIV infection, showing a positive attitude towards the use of NO-PEP. High numbers reported that it should be used for rape cases and in cases where piercing objects and needles are shared. A high number of students felt that NO-PEP should be given for consensual unprotected sex. This could mean that the students engage in risky sexual behaviour, and warrants further investigation. Investigations also need to be done to explore why most students felt NO-PEP should not be given for people sharing razors.

Two-thirds of the students thought that it is important to use NO-PEP, yet only 28% reported knowledge of NO-PEP. Further studies need to be done to explore why students think NO-PEP is important whilst they do not know what NO-PEP is, and why some students consider NO-PEP to be unimportant. A large number of students felt that it was beneficial to learn about NO-PEP. Preventing HIV infection, seeking early medical help, protecting a partner, and protecting an unborn baby were stated as benefits to learning about NO-PEP. However, substantial numbers did not think it beneficial to learn about NO-PEP. Inappropriately, a large number of students thought that testing for HIV and being on NO-PEP are beneficial to act as proxy diagnosis for a partner.

In identifying barriers to access to NO-PEP, most students knew where it can be accessed. It was noted that most students reported that they would get NO-PEP from private hospitals as opposed to getting it from the campus clinic. This could implicate fear of discrimination from their friends on campus and healthcare workers, as these were specifically mentioned as barriers. Further investigations need to be done to verify this assumption. Students reported discrimination (by friends, colleagues, employers, family and healthcare workers), being neglected by partner and friends, rejection by family and poor accessibility to PEP services as barriers to the access to and use of NO-PEP. These perceived barriers, as anticipated, are probably related to stigmatisation around HIV-related issues. These barriers could be addressed if efforts were made to reduce stigmatisation, and this is beyond the scope of this study. Students felt that discussing the following were not barriers to the use of NO-PEP: condom use, not sharing needles, not doing drugs, and going for HIV testing.

There was a low response rate on questions relating to knowledge of medication used for NO-PEP and precautions to be taken whilst on treatment. This could be due to these students being in their preclinical years and not having been taught on such. The lack of knowledge in this sample of students could, however, mean that knowledge on NO-PEP in the general population is low. Further investigations are needed for clarification. When measuring associations between self-reported and objective knowledge on medication used for PEP, students only knew what needs to be done if a person on NO-PEP forgets a dose. However, some trends were observed indicating that self-reporting may be an appropriate tool to measure knowledge; this should be confirmed in further studies. On precautions to be taken whilst on NO-PEP, the high non-response rate is worrying, as one would expect this group to know. Even amongst those who responded, there were substantial numbers that did not know. This high non-response rate needs to be explored in more detail.

### Strengths and limitations

This study is the first of its kind at this campus involving medical undergraduates, and thus provides valuable information. However, the sample comprised mostly first- and second-year students, as the numbers of students from third to final year that participated were not representative of the years and were excluded from analyses. For this reason these findings cannot be generalised to all medical students. There also could have been measurement biases, whereby we over-report on students’ knowledge. This is because the questionnaire had answer options displayed below each question which participants had to choose from, i.e. the questions were not open-ended. It is thus unclear whether participants actually had this knowledge on NO-PEP or whether recognition of the options artificially increased the reported knowledge levels. The questionnaire used for the study was developed from one used by another author and modified to meet the objectives of this study, and this could have resulted in unclear questions.

## Conclusion and recommendations

This was an exploratory study to obtain some information on knowledge and attitudes towards NO-PEP in this particular population, in order to get some baseline information on this topic and to evaluate simple associations existing in the dataset. Findings from this study show that knowledge of HIV infection prevention and transmission is good amongst this group of students, although there are still concerns on the numbers that lack knowledge. Knowledge on NO-PEP is poor and this shows the need to improve educational programmes in life sciences in schools, in healthcare facilities, in communities and the media to increase awareness.

Findings from this study also show that there is stigmatisation around the use of NO-PEP, as shown by the barriers identified by the students. Increasing awareness through the above-mentioned measures could potentially reduce this stigmatisation and ultimately reduce new HIV infections. Information gathered from this study could provide insight on whether medical students are being equipped with the necessary information pertaining to NO-PEP. Hence this study could potentially influence policy regarding teaching practices on HIV infection treatment and prevention. Further studies should investigate issues as highlighted in the discussion. Investigations should also be done to assess whether the level of knowledge is different between the various years of study.

## References

[CIT0001] World Health Organization Media Centre HIV/AIDS. WHO/HIV/AIDS [homepage on the Internet]. No date [cited 2012 Nov 15]. Available from: http://www.who.org

[CIT0002] Orisakwe EE, Ross AJ, Ocholla PO Correlation between knowledge of HIV, attitudes and perceptions of HIV and a willingness to test for HIV at a regional hospital in KwaZulu-Natal, South Africa. Afr J Prm Hlth Care Fam Med. 2012;4(1):8.

[CIT0003] Dube NM, Summers R, Tint KS, Mayayise G A pharmacologivilance study of adults on highly active antiretroviral therapy, South Africa: 2007–2011. Pan Afr Med J. 2012;11:39. 22593775 PMC3343667

[CIT0004] Hamers RL, Sigaloff KC, Wensing AM, Wallis CL, Kityo C, Siwale M, Mandaliya K, Ive P, Botes ME, Wellington M, Osibogun A, Stevens WS, Rinke de Wit TF, Schuurman R. PharmAccess African Studies to Evaluate Resistance (PASER) Patterns of HIV-1drug resistance after first-line antiretroviral therapy (ART) failure in 6 sub-Saharan African countries: implications for second-line ART strategies. Clin Infect Dis. 2012;54(11):1660–1669. http://dx.doi.org/10.1093/cid/cis254 22474222 10.1093/cid/cis254

[CIT0005] World Health Organization Department of HIV/AIDS Prevention and treatment of HIV and other sexually transmitted infections for sex workers in low- and middle-income countries. Recommendations for a public health approach [homepage on the Internet]. No date [cited 2012 Oct 1]. Available from: http://www.who.int/hiv/pub/guidelines/en/26131541

[CIT0006] Kathmandu T National anti-retroviral therapy guidelines [homepage on the Internet]. 2009 [cited 2012 Nov 30]. Available from: http://www.who.int/search?q=hiv+firstaid+pep

[CIT0007] World Health Organization HIV post exposure prophylaxis for occupational and non-occupational exposure to HIV [homepage on the Internet]. No date [cited 2012 Dec 2]. Available from: http://www.who.int/hiv/topics/prophylaxis/meeting/en/index.html

[CIT0008] Minas B, Laing S, Jordan H, Mak DB Improved awareness and appropriate use of non-occupational post-exposure prophylaxis (nPEP) for HIV prevention following a multi-modal communication strategy. BMC Public Health. 2012;12:906 http://dx.doi.org/10.1186/1471-2458-12-906 23095456 10.1186/1471-2458-12-906 PMC3503851

[CIT0009] Western Cape Provincial Department of Health Treatment guidelines for the use of post-exposure prophylaxis (PEP) for the prevention of the transmission of human immunodeficiency virus (HIV) in males and females who have been raped or sexually assaulted [homepage on the Internet]. 2002 [cited 2012 Nov 30]. Available from: http://www.doh.gov.za/aids/docs/rape-protocol.html

[CIT0010] Kim JC, Martin LJ, Denny L Rape and HIV post-exposure prophylaxis: Addressing the dual epidemics in South Africa. Reprod Health Matters. 2003;11(22):101–112. http://dx.doi.org/10.1016/S0968-8080(03)02285-7 14708401 10.1016/s0968-8080(03)02285-7

[CIT0011] Laporte A, Jourdan N, Bouvet E, Lamontagne F, Pillonel J, Desenclos J Post-exposure prophylaxis after non-occupational HIV exposure: Impact of recommendations on physicians’ experiences and attitudes. AIDS. 2002; 16(3):397–405. http://dx.doi.org/10.1097/00002030-200202150-00011 11834951 10.1097/00002030-200202150-00011

[CIT0012] Rey D, Bouhnik AD, Peretti-Watel P, Obadia Y, Spire B Awareness of non-occupational HIV postexposure prophylaxis among French people living with HIV: The need for better targeting. AIDS 2007; 21:S71–S76. http://dx.doi.org/10.1097/01.aids.0000255088.44297.26 10.1097/01.aids.0000255088.44297.26 17159591

[CIT0013] Mehta SA, Silvera R, Bernstein K, Holzman RS, Aberg JA, Daskalakis DC Awareness of post-exposure HIV prophylaxis in high-risk men who have sex with men in New York City. Sex Transm Infect. 2011; 87(4):344–348. http://dx.doi.org/10.1136/sti.2010.046284 21357600 10.1136/sti.2010.046284

[CIT0014] Hayter M Knowledge and attitudes of nurses working in sexual health clinics in the united kingdom toward post-sexual exposure prophylaxis for HIV infection. Public Health Nursing. 2004; 21(1):66–72. http://dx.doi.org/10.1111/j.1525-1446.2004.21109.x 14692991 10.1111/j.1525-1446.2004.21109.x

[CIT0015] Giele C, Maw R, Carne C, Evans B Post-exposure prophylaxis for non-occupational exposure to HIV: Current clinical practice and opinions in the UK. Sex Transm Infect. 2002; 78(2):130–132. http://dx.doi.org/10.1136/sti.78.2.130 12081175 10.1136/sti.78.2.130 PMC1744438

[CIT0016] Roland ME, Myer L, Martin LJ, et al. Preventing human immunodeficiency virus infection among sexual assault survivors in Cape Town, South Africa: An observational study. AIDS Behav. 2012; 16(4):990–998. http://dx.doi.org/10.1007/s10461-011-9892-3 21301949 10.1007/s10461-011-9892-3 PMC3337999

[CIT0017] Kim JC, Askew I, Muvhango L, et al. Comprehensive care and HIV prophylaxis after sexual assault in rural South Africa: The Refentse intervention study. BMJ 2009;338:b515 http://dx.doi.org/10.1136/bmj.b515 19286746 10.1136/bmj.b515

[CIT0018] Levy I Post exposure prophylaxis (PEP) to prevent HIV after sexual exposure in Israel. Harefuah. 2005;144(4):252–254. 15889608

[CIT0019] Chogle NL, Chogle MN, Divatia JV, Dasgupta D Awareness of post-exposure prophylaxis guidelines against occupational exposure to HIV in a Mumbai hospital. Natl Med J India. 2002; 15(2):69–72. 12044118

[CIT0020] Fadeyi A, Fowotade A, Abiodun MO, Jimoh AK, Nwabuisi C, Desalu OO Awareness and practice of safety precautions among healthcare workers in the laboratories of two public health facilities in Nigeria. Niger Postgrad Med J. 2011; 18(2):141–146. 21670783

[CIT0021] Liu AY, Kittredge PV, Vittinghoff E, et al. Limited knowledge and use of HIV post- and pre-exposure prophylaxis among gay and bisexual men. J Acquir Immune Defic Syndr. 2008; 47(2):241–247. http://dx.doi.org/10.1097/QAI.0b013e31815e4041 18340656

[CIT0022] Western Cape Provincial Department of Health Tygerberg Hospital: Overview [homepage on the Internet]. April 2014 [cited 2014 May 29]. Available from: www.westerncape.gov.za/your_gov/153

[CIT0023] Stellenbosch University Faculty of Medicine and Health Sciences 2013 Health Sciences Yearbook. 2013 [cited 2014 May 29]. Available from: http://sun.ac.za/english/Documents/Yearbooks/2013/2013_WEB_Medicine_and_Health%20Sciences_25Jan2013.pdf

